# Glyceraldehyde-3-Phosphate Dehydrogenase Acts as a Mitochondrial Trans-*S*-Nitrosylase in the Heart

**DOI:** 10.1371/journal.pone.0111448

**Published:** 2014-10-27

**Authors:** Mark J. Kohr, Elizabeth Murphy, Charles Steenbergen

**Affiliations:** 1 Department of Pathology, Johns Hopkins University School of Medicine, Baltimore, Maryland, United States of America; 2 Systems Biology Center, National Heart Lung and Blood Institute/National Institutes of Health, Bethesda, Maryland, United States of America; David Geffen School of Medicine at UCLA, United States of America

## Abstract

Mitochondrial proteins have been shown to be common targets of *S*-nitrosylation (SNO), but the existence of a mitochondrial source of nitric oxide remains controversial. SNO is a nitric oxide-dependent thiol modification that can regulate protein function. Interestingly, trans-*S*-nitrosylation represents a potential pathway for the import of SNO into the mitochondria. The glycolytic enzyme glyceraldehyde-3-phosphate dehydrogenase (GAPDH), which has been shown to act as a nuclear trans-*S*-nitrosylase, has also been shown to enter mitochondria. However, the function of GAPDH in the mitochondria remains unknown. Therefore, we propose the hypothesis that *S*-nitrosylated GAPDH (SNO-GAPDH) interacts with mitochondrial proteins as a trans-*S*-nitrosylase. In accordance with this hypothesis, SNO-GAPDH should be detected in mitochondrial fractions, interact with mitochondrial proteins, and increase mitochondrial SNO levels. Our results demonstrate a four-fold increase in GAPDH levels in the mitochondrial fraction of mouse hearts subjected to ischemic preconditioning, which increases SNO-GAPDH levels. Co-immunoprecipitation studies performed in mouse hearts perfused with the *S*-nitrosylating agent S-nitrosoglutathione (GSNO), suggest that SNO promotes the interaction of GAPDH with mitochondrial protein targets. The addition of purified SNO-GAPDH to isolated mouse heart mitochondria demonstrated the ability of SNO-GAPDH to enter the mitochondrial matrix, and to increase SNO for many mitochondrial proteins. Further, the overexpression of GAPDH in HepG2 cells increased SNO for a number of different mitochondrial proteins, including heat shock protein 60, voltage-dependent anion channel 1, and acetyl-CoA acetyltransferase, thus supporting the role of GAPDH as a potential mitochondrial trans-*S*-nitrosylase. In further support of this hypothesis, many of the mitochondrial SNO proteins identified with GAPDH overexpression were no longer detected with GAPDH knock-down or mutation. Therefore, our results suggest that SNO-GAPDH can act as a mitochondrial trans-*S*-nitrosylase, thereby conferring the transfer of SNO from the cytosol to the mitochondria.

## Introduction

Redox-dependent protein modifications are quickly emerging as important regulators of many different cellular processes, including mitochondrial function [1], [2]. *S*-nitrosylation (SNO) in particular, has been identified as a critical regulator of a diverse array of cellular signaling pathways. SNO is a reversible, nitric oxide (NO)-dependent thiol modification that has been shown to modulate protein function [3]–[6], stabilize protein levels [7], [8], mediate protein-protein interaction [9]–[11], and alter the localization of target proteins [9], [12]. SNO has also been shown to play a critical role in protecting cysteine residues from irreversible oxidation [3], [13]. In recent studies, we have demonstrated that myocardial ischemic preconditioning (IPC) induces a robust increase in protein SNO that correlates with cardioprotection [3], [4], [13]. Common SNO targets included the glycolytic enzyme glyceraldehyde-3-phosphate dehydrogenase (GAPDH) and many different mitochondrial proteins. NO is a well-known regulator of mitochondrial respiration [1], [2], and SNO has been shown to modulate the activity of various components of the electron transport chain, including complex I [5] and the F_1_F_0_-ATPase [4], [14]. However, the source for NO within the mitochondria has not been clearly defined, and the existence of a mitochondrial isoform of NO synthase remains controversial [15].

SNO is initiated through several different pathways, which include direct thiol modification by an NO derivative (i.e., NO^+^, N_2_O_3_). Trans-*S*-nitrosylation represents an additional pathway for the formation of SNO and is achieved by the action of trans-*S*-nitrosylases which serve to directly transfer the NO moiety to a recipient protein. As a result, trans-*S*-nitrosylases serve to propagate SNO beyond local NO signaling domains. Therefore, trans-*S*-nitrosylases represent a potential mechanism for the import of SNO into the mitochondria [16]. Interestingly, SNO has been shown to initiate the nuclear translocation of GAPDH in neurons [9], where GAPDH can act as a nuclear trans-*S*-nitrosylase and regulate gene transcription [17]. GAPDH has also been shown to enter mitochondria [18], [19], and may regulate mitochondrial membrane potential [19]. Although a putative mitochondrial import sequence has been identified [19], the ability of *S*-nitrosylated GAPDH (SNO-GAPDH) to enter the mitochondrial matrix, as well as a potential role for GAPDH as a mitochondrial trans-*S*-nitrosylase, has not been investigated.

In the current study, we sought to define a role for GAPDH in the mitochondrial import of SNO. We tested the hypothesis that SNO-GAPDH interacts with new mitochondrial protein targets, which would facilitate trans-*S*-nitrosylation. Herein, we provide evidence that myocardial IPC, which increases global SNO-GAPDH levels [3], also increases GAPDH levels in the mitochondrial fraction. We also provide evidence that SNO-GAPDH can gain entry into the mitochondrial matrix. Further, we demonstrate that SNO-GAPDH interacts with new mitochondrial protein targets, resulting in trans-*S*-nitrosylation of mitochondrial proteins.

## Materials and Methods

### Animals

Male C57BL/6 mice were obtained from Jackson Laboratories (Bar Harbor, ME). All animals utilized in this study were between the ages of 12–15 weeks. Mice were anesthetized with pentobarbital sodium (50–100 mg/kg) via intraperitoneal injection in order to minimize pain and distress during all procedures. This investigation conforms to the *Guide for the Care and Use of Laboratory Animals* published by the US National Institutes of Health (NIH publication No. 85-23, revised 2011) and was approved by the Institutional Laboratory Animal Care and Use Committee at Johns Hopkins University and the National Heart, Lung and Blood Institute.

### Solutions and drugs

Krebs-Henseleit buffer consisted of (in mmol/L): NaCl (120), KCl (4.7), KH_2_PO_4_ (1.2), NaHCO_3_ (25), MgSO_4_ (1.2), D-Glucose (11), and CaCl_2_ (1.75); pH 7.4. Krebs-Henseleit buffer was bubbled with 95% O_2_/5% CO_2_. *S*-nitrosoglutathione (GSNO; Sigma, St. Louis, MO) was used as an *S*-nitrosylating agent. All solutions were made fresh on the day of experimentation.

### Perfusion protocols and mitochondrial isolation

**Figure 1:**
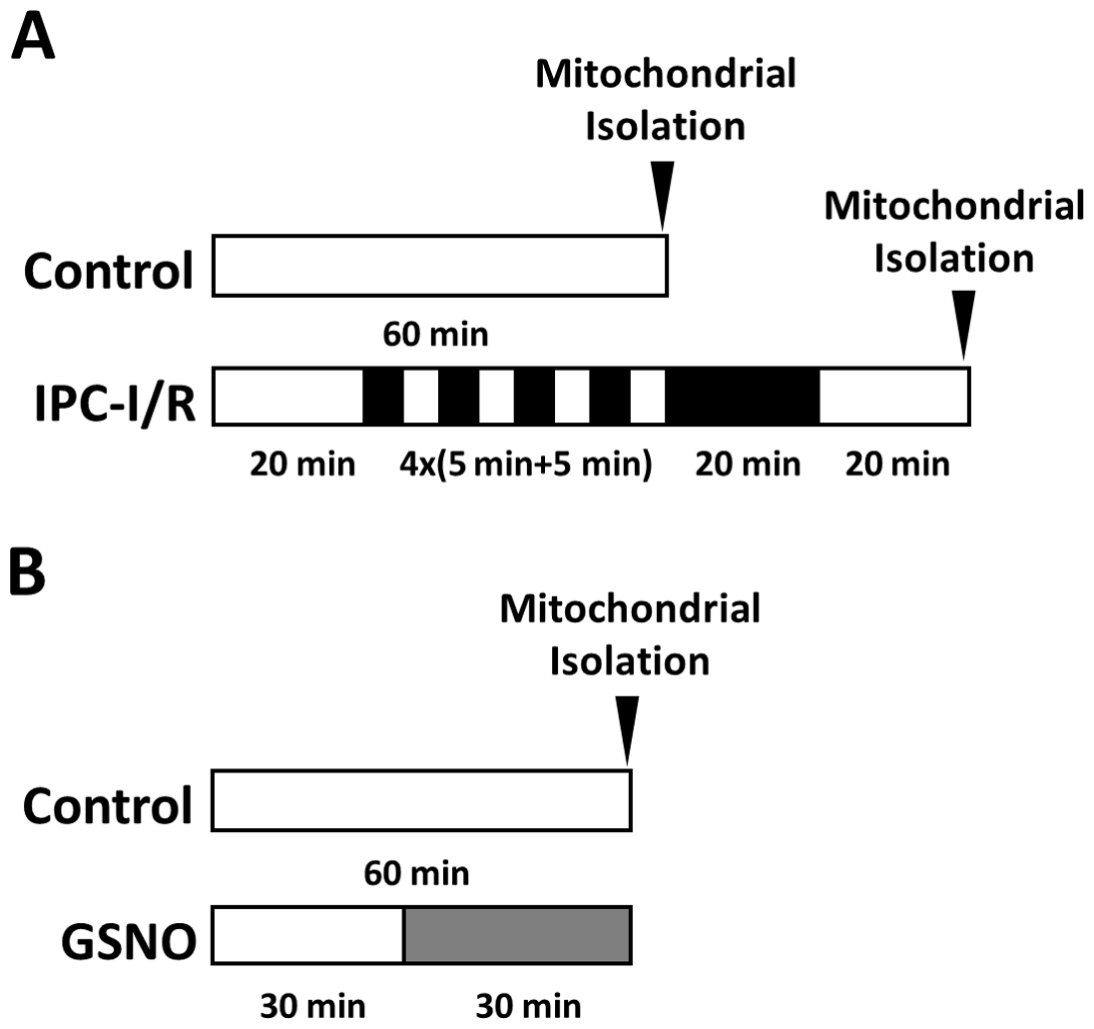
Myocardial perfusion protocols. Perfusion protocols for Figure 2 (**A**) and Table 1 (**B**).

Hearts were Langendorff-perfused in the dark as previously described [3], [4], [13], [20], [21], and randomly subjected to a perfusion protocol. For analysis of mitochondrial GAPDH levels (Figure 1a), hearts were subjected to control perfusion (60 minutes perfusion) or an IPC-ischemia/reperfusion protocol (IPC-IR; 20 minutes perfusion, 4 cycles of 5 minutes ischemia/5 minutes reperfusion, 20 minutes ischemia, 20 minutes reperfusion). For analysis of GAPDH binding partners (Figure 1b), hearts were subjected to control perfusion (60 minutes perfusion) or a GSNO protocol (30 minutes perfusion, 30 minutes GSNO [100 µmol/L]). Heart mitochondria were then isolated using differential centrifugation as previously described [6], [22]. Briefly, hearts were minced, homogenized in mitochondrial isolation buffer B containing (in mmol/L): mannitol (225), sucrose (75), MOPS (5), taurine (2), EGTA (1), EDTA (1), Neocuproine (0.1); pH 7.25 with protease/phosphatase inhibitors, and digested with trypsin (0.0001 g/0.1 g tissue; Sigma) for 5 minutes at 4°C. The homogenate was then centrifuged at 500×g, and the resulting supernatant was centrifuged at 11,000×g. The resulting pellet was then recovered as the mitochondrial fraction, and the supernatant was recovered as the cytosolic fraction. For HepG2 mitochondrial isolation, cells were removed from the cell culture dish and rinsed with PBS. HepG2 mitochondria were then isolated using the same protocol described above, with the exception that HepG2 cell lysate was not subjected to trypsin digestion.

### Quantification of mitochondrial GAPDH from perfused myocardium

Following mitochondrial isolation, samples were separated by one-dimensional sodium dodecyl sulfate (SDS)-polyacrylamide gel electrophoresis. The resulting gel was stained with coomassie blue stain overnight at room temperature. The band corresponding to GAPDH (37 kDa) was then excised from the gel and the gel pieces were destained in 25 mmol/L NH_4_HCO_3_/50% methanol. Gel pieces were then dehydrated in 25 mmol/L NH_4_HCO_3_/50% acetonitrile, dried using a SpeedVac (Thermo), and subjected to reduction and alkylation in 10 mmol/L dithiothreitol (in 25 mmol/L NH_4_HCO_3_) at 56°C for 1 hour, followed by 55 mmol/L iodoacetamide (in 25 mmol/L NH_4_HCO_3_) at room temperature in the dark for 1 hour. Gel pieces were then dehydrated, dried, and subjected to in-gel trypsin digestion (Promega, Madison, WI) in 50 mmol/L NH_4_HCO_3_ at 4°C for 10 minutes followed by overnight incubation at 37°C. Peptides were then extracted from the gel pieces in 0.1% formic acid/50% acetonitrile, dried, and resuspended in 0.1% formic acid and cleaned with a C_18_ column (ZipTip; Millipore, Billerica, MA). Liquid chromatography-tandem mass spectrometry (LC-MS/MS) was then performed using an LTQ Orbitrap Velos mass spectrometer (Thermo, San Jose, CA) as previously described [3], [21]. Relative quantification of GAPDH was performed using QUOIL (Quantification withOut Isotope Labeling), an in-house software program designed as a label-free approach to peptide quantification [23].

### Incubation of GAPDH with isolated mitochondria

Purified GAPDH or SNO-GAPDH was incubated with mitochondria isolated from control hearts (10 minutes perfusion), IPC hearts (20 minutes perfusion, 4 cycles of 5 minutes ischemia/5 minutes reperfusion), or HepG2 cells for 30 minutes at room temperature. Isolated mitochondria were then subjected to trypsin digestion (100 µg/mL; Sigma) for 10 minutes at 4°C; any protein that remains outside of the mitochondria will be digested, while proteins in the mitochondrial matrix will be protected from trypsin digestion. Western blot was used to confirm GAPDH import into the mitochondria. TOM20 is an outer mitochondrial protein that was used as a marker for trypsin digestion, and the F_1_F_0_-ATPase is a protein that is found within the mitochondrial matrix and was used as a protein loading control. SNO-GAPDH was generated by incubating purified GAPDH with 200 µmol/L GSNO for 30 minutes at room temperature; excess GSNO was removed via Zeba desalting columns (Thermo).

### GAPDH co-immunoprecipitation from perfused myocardium

Following cytosolic and mitochondrial isolation, samples were incubated overnight at 4°C with a GAPDH antibody-coupled agarose resin (Santa Cruz, Dallas, TX) in HEN buffer containing (in mmol/L): **H**EPES-NaOH 7.8 (250), **E**DTA (1), and **N**eocuproine (0.1) with 2.5% SDS (w/v) and an EDTA-free protease inhibitor tablet (Roche, Indianapolis, IN); IgG-coupled agarose resin was used as a negative control. Captured proteins were washed with HEN buffer, eluted from the resin in 50% acetonitrile/0.4% trifluoroacetic acid, and dried using a SpeedVac (Thermo). Samples were then resuspended and subjected to trypsin digestion (Promega) overnight at 37°C with rotation in buffer containing 50 mmol/L NH_4_HCO_3_ and 1 mmol/L EDTA, and dried. Samples were then resuspended in 0.1% formic acid, and cleaned with a C_18_ column (ZipTip; Millipore). LC-MS/MS was then performed using an LTQ Orbitrap Velos mass spectrometer (Thermo) as previously described [3], [21].

### DyLight maleimide switch and one-dimensional gel electrophoresis

A modified version of the biotin switch was performed as previously described [4], [24]. Briefly, samples were diluted in HEN buffer with 2.5% SDS and treated with 50 mmol/L *N*-ethylmaleimide (NEM; Sigma) for 20 minutes at 50°C with gentle vortexing every 5 minutes in order to block free thiol groups from modification; NEM was removed via acetone precipitation. SNO cysteines were then reduced with ascorbate (1 mmol/L) and labeled with a DyLight maleimide fluor (Thermo). Samples were then separated via one-dimensional SDS-polyacrylamide gel electrophoresis and imaged using a LiCor Odyssey scanner (Lincoln, NB).

### HepG2 culture and transfection

HepG2 cells (ATCC, Manassas, VA) were maintained in Dulbecco's Modified Eagle Medium supplemented with 10% fetal bovine serum and 1% penicillin/streptomycin (Life Technologies). DDK-tagged human GAPDH was purchased from Origene (Rockville, MD), and mutation of C150 to a serine residue was performed using the QuickChange II site-directed mutagenesis kit (Agilent, Santa Clara, CA) according to the manufacturer's instruction. GAPDH-DDK or GAPDH_C150S_-DDK or a GFP control vector (pMax-GFP; Lonza, Walkersville, MD) was transfected into HepG2 cells using XtremeGene DNA HP (Roche) according to the manufacturer's instruction; cells were incubated with the vector/transfection mixture for 48 hours. GAPDH siRNA (100 pmol; Life Technologies) or a siRNA scramble (Silencer Select negative control 1; Life Technologies) was transfected into HepG2 cells using electroporation (Amaxa Nucleofector; Lonza) according to the manufacturer's instruction. Sodium pyruvate (1 mmol/L) was added to the culture medium following transfection.

### Western blotting

Samples were run on a 4–12% gel and transferred onto a nitrocellulose membrane. Membranes were blocked with 5% milk in Tris-buffered saline with 0.1% Tween-20 and subsequently incubated with primary antibodies against GAPDH (1∶1,000; Santa Cruz), TOM20 (1∶1,000; Santa Cruz), F_1_F_0_-ATPase α subunit (1∶50,000; Abcam, Cambridge, MA), neuronal NO synthase (1∶1,000; Santa Cruz), endothelial NO synthase (1∶1,000; Santa Cruz), β-actin (1∶1,000, Abcam), or enolase (1∶1,000, Santa Cruz) overnight at 4°C. Membranes were then probed with the corresponding secondary antibodies for 1 hour and visualized by electrogenerated chemiluminescence (GE Healthcare, Piscataway, NJ). Densitometry was assessed using ImageJ software (NIH, Bethesda, MD).

### S-nitrosylation-resin assisted capture (SNO-RAC)

HepG2 cells were lysed in HEN buffer containing 1.0% (w/v) triton X-100 and 20 mmol/L NEM. SNO-RAC was then used to examine protein SNO as previously described [3], [21]. Briefly, samples were diluted in HEN buffer with 2.5% SDS and treated with 50 mmol/L NEM (Sigma) for 20 minutes at 50°C with gentle vortexing every 5 minutes; NEM was removed via acetone precipitation. SNO proteins were then reduced with ascorbate (20 mmol/L), captured with thiopropyl sepharose (GE), and digested with trypsin (Promega). The resulting peptides were eluted using 20 mmol/L dithiothreitol (Thermo). LC-MS/MS was then performed using an LTQ Orbitrap Velos mass spectrometer (Thermo) as previously described [3], [21].

### Liquid chromatography-tandem mass spectrometry analysis

LC-MS/MS raw files were analyzed using Proteome Discoverer 1.3 (Thermo) with the NIH six-processor MASCOT cluster search engine (http://biospec.nih.gov, version 2.3). The following search criteria were used: database, Swiss-Prot (Swiss Institute of Bioinformatics); taxonomy, Homo sapiens (human); enzyme, trypsin; miscleavages, 3; variable modifications, oxidation (M), *N*-ethylmaleimide (C), deamidation (NQ); MS peptide tolerance 25 ppm; MS/MS tolerance as 0.8 Da. Peptides were filtered at a false discovery rate of 1%, as determined by a targeted decoy database search with a significance threshold of 0.03. Proteome Discoverer 1.3 was also used for label-free peptide quantification analysis.

### Statistics

Data are presented as mean ± SEM. Statistical significance (p<0.05) was determined between groups using an ANOVA followed by a Tukey post-hoc test for multiple groups or a Student's t-test for two groups.

## Results

### Myocardial IPC increases GAPDH levels in the mitochondrial fraction

**Figure 2:**
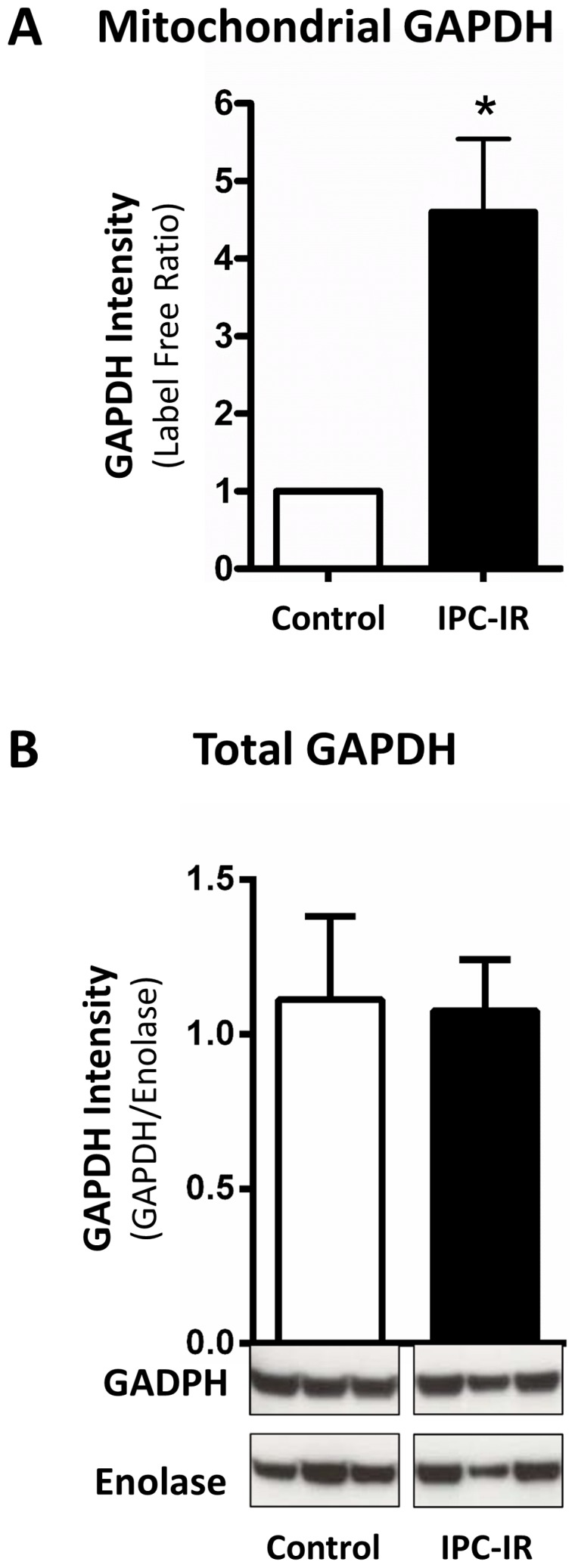
Mitochondrial GAPDH and total GAPDH levels following cardioprotection. (**A**) GAPDH protein levels in the mitochondrial fraction of hearts subjected to control or an IPC-I/R protocol were assessed via liquid chromatography-tandem mass spectrometry, followed by label-free peptide quantification (*p<0.05 vs. control, n = 3). (**B**) Total GAPDH protein levels in hearts subjected to control or an IPC-I/R protocol were assessed via western blot analysis. Representative western blots are shown for total GAPDH (upper) and enolase (lower) in whole heart homogenates together with the densitometry of GAPDH normalized to enolase (n = 3).

In previous studies, we demonstrated that myocardial IPC induces a robust increase in protein SNO, and common targets included cytosolic GAPDH and many mitochondrial proteins [3], [4]. Although NO has a high rate of diffusion, NO is also very reactive and thus has limited bioavailability. As a result, the mechanism(s) by which NO is targeted to the mitochondria is not known. One way that mitochondrial protein SNO could occur is through the translocation of SNO-GAPDH to the mitochondria. Therefore, to test our hypothesis that GAPDH acts as a mitochondrial trans-*S*-nitrosylase, we examined mitochondrial fractions from control hearts and IPC hearts subjected to ischemia/reperfusion injury (IPC-IR; Figure 1a), for evidence of mitochondrial GAPDH using mass spectrometry. GAPDH showed a four-fold increase in the mitochondrial fraction of hearts subjected to IPC-IR compared to control hearts, as determined via label-free peptide quantification (Figure 2a), while total GAPDH levels remained unchanged as assessed via western blot (Figure 2b). Mitochondrial SNO-GAPDH levels were not directly quantified because extensive mitochondrial purification (i.e., percoll gradient, etc.) would be required to effectively separate the cytosolic SNO-GAPDH from the mitochondrial SNO-GAPDH, and given the labile nature of *S*-nitrosylation, the modification is likely to be lost during this lengthy procedure. Additionally, since the mitochondria were isolated via differential centrifugation, it is also possible that GAPDH co-fractionates with the mitochondria. Nonetheless, this result supports the ability of GAPDH to potentially act as a mitochondrial trans-*S*-nitrosylase, thus leading us to further pursue this hypothesis.

### GAPDH is imported into heart mitochondria

**Figure 3:**
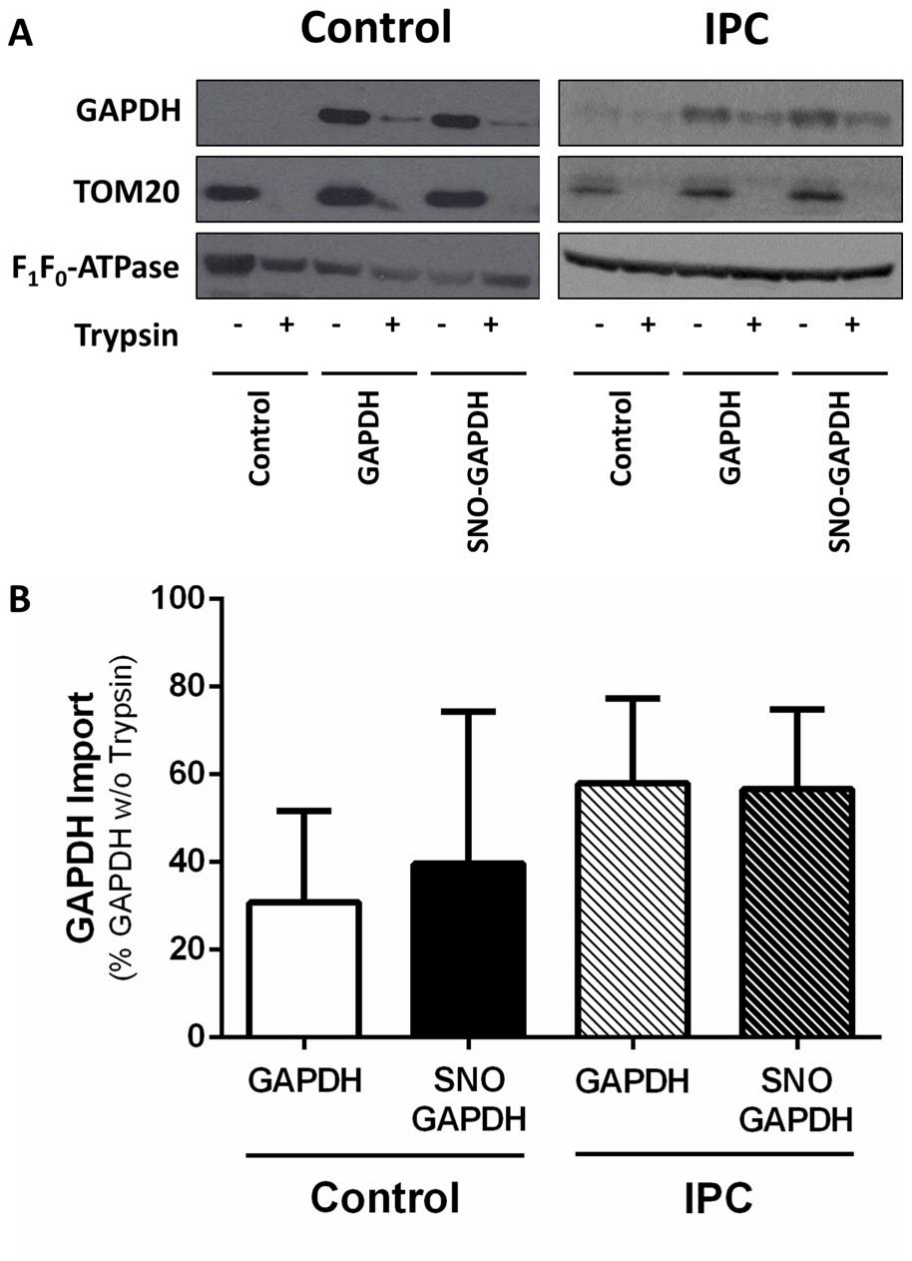
GAPDH and SNO-GAPDH are imported into the matrix of heart mitochondria. Mitochondrial GAPDH protein levels were assessed after the addition of purified GAPDH or SNO-GAPDH to isolated mitochondria from control and IPC hearts. (**A**) Representative western blots for GAPDH (upper), TOM20 (center), and the α subunit of F_1_F_0_-ATPase (lower) in control heart and IPC heart mitochondria. Control: non-treated mitochondrial control; GAPDH: purified GAPDH treated mitochondria; SNO-GAPDH: SNO-GAPDH treated mitochondria. (**B**) GAPDH import into control and IPC heart mitochondria as assessed via the percentage of GAPDH following trypsin digestion compared to GAPDH levels prior to the addition of trypsin. GAPDH levels were assessed via densitometry (n = 3).

GAPDH has been shown to associate with mitochondria in various studies [18], [19], but the ability of SNO-GAPDH to enter the mitochondria has not been examined. Therefore, we next performed experiments to determine if SNO-GAPDH can be imported into the matrix of mitochondria isolated from control hearts and hearts subjected to IPC. Purified GAPDH and SNO-GAPDH were separately incubated with isolated heart mitochondria for 1 hour; mitochondria were then subjected to trypsin digestion (trypsin digests proteins outside of the mitochondrial membrane without affecting proteins within the mitochondria). The outer mitochondrial protein TOM20 was used as a positive control for trypsin digestion, while the inner mitochondrial protein F_1_F_0_-ATPase was used as a negative control. Following the addition of purified GAPDH to mitochondria isolated from either control or IPC hearts, the majority of the protein was degraded by trypsin, but there was a small amount that remained for both groups and this is indicative of GAPDH entry into mitochondria (Figure 3a). SNO-GAPDH was also detected following trypsin digestion for control and IPC heart mitochondria, and both GAPDH and SNO-GAPDH appeared to be imported into the mitochondria equally well (Figure 3b). In addition, the amount of GAPDH or SNO-GAPDH that was imported into the mitochondria was not different between control and IPC hearts (Figure 3b). Although there appears to be a trend towards increased GAPDH import into the mitochondria, this difference is not significant and likely results from the IPC-induced mitochondrial import of endogenous GAPDH. These data are consistent with a previous study [19], and demonstrate the ability of SNO-GAPDH to enter the mitochondrial matrix.

### GSNO induces the interaction of GAPDH with mitochondrial protein targets

**Figure 4:**
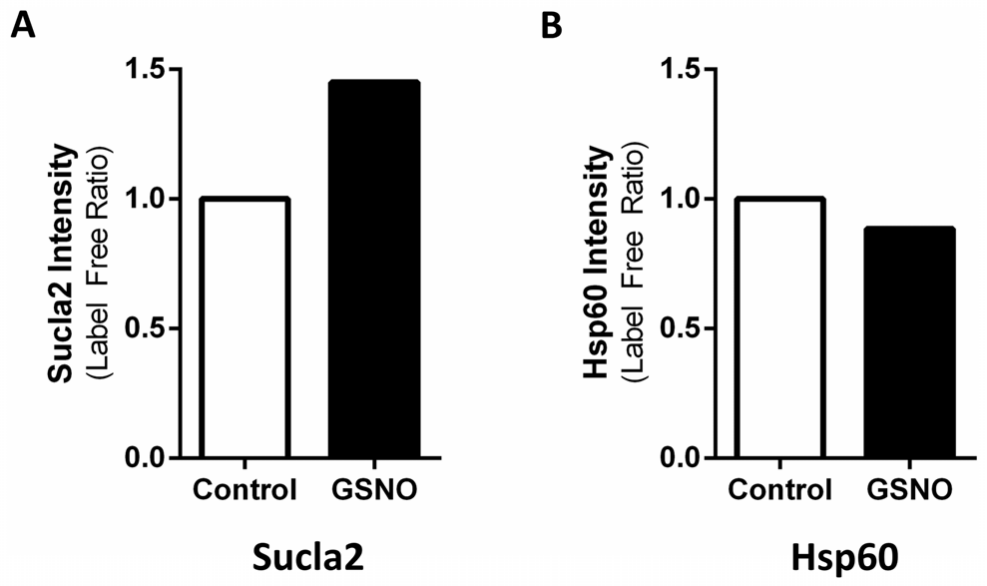
SNO alters the binding of GAPDH to Sucla2, but not Hsp60. Sucla2 (**A**) and Hsp60 (**B**) levels from GAPDH co-immunoprecipitation using mitochondrial fractions of hearts subjected to control or GSNO perfusion. Binding was assessed using label-free peptide quantification (n = 6).

SNO has been shown to alter the binding partners of target proteins, thus modulating protein-protein interaction [9]–[11]. Therefore, to further test our hypothesis, we performed experiments to examine the effect of SNO on the binding partners of GAPDH in cytosolic and mitochondrial fractions using co-immunoprecipitation in tandem with mass spectrometry. Perfusion of the myocardium with the *S*-nitrosylating agent GSNO (Figure 1b), which we have shown to target GAPDH [21], led to a shift in cytosolic and mitochondrial GAPDH binding partners (Table 1). Under control conditions, GAPDH tended to interact with different proteins in the cytosol, including actin and tubulin, but we observed very little interaction between GAPDH and any mitochondrial proteins. However, following perfusion with GSNO, GAPDH interacted with new mitochondrial protein targets, including aconitate hydratase, OPA1, and the mitochondrial phosphate carrier. We further assessed whether SNO altered the binding of GAPDH to various targets by analyzing the common mitochondrial binding partners of GAPDH (+/− GSNO) using label-free peptide quantification. This approach allowed us to determine the amount of each target protein that was pulled down with GAPDH. The results of this analysis suggest that GAPDH binding may be increased with SNO for some mitochondrial binding partners, including succinyl CoA ligase (Sucla2; Figure 4a), but remain unchanged for other mitochondrial target proteins, such as heat shock protein 60 (Hsp60; Figure 4b). However, additional studies are necessary to make a definitive conclusion with regard to the effect of SNO on GAPDH binding affinity. Regardless of the effect of SNO on GAPDH binding, these results demonstrate a clear shift in the binding partners of GAPDH following SNO.

**Table 1:** GAPDH binding partners in cytosolic and mitochondrial fractions of hearts subjected to control or GSNO perfusion.

*Cytosolic Fraction*			*Mitochondrial Fraction*		
Common IDs	Unique IDs Control	Unique IDs GSNO	Common IDs	Unique IDs Control	Unique IDs GSNO
Elongation Factor 1-α1	Actin	cAMP-dependent protein kinase catalytic subunit α	60 kDa heat shock protein		Aconitate hydratase
Tubulin β-2C chain	Alpha-crystallin B	Transformer-2 protein homolog α	Succinyl-CoA ligase [ADP-forming] β		Creatine kinase, S
	Troponin I	tRNA wybutosin-synthesizing protein 3 homolog			Cytochrome C oxidase 6C
	Troponin T				Dynamin-like 120 kDa
	Tubulin-α-1C		*Non-mitochondrial IDs:*	*Non-mitochondrial IDs:*	Elongation factor Tu
	Tubulin β-6		Myosin-6	Myosin regulatory light chain 2	Isocitrate dehydrogenase [NAD] α
	Tubulin β-4			Myosin4	Phosphate carrier

LC-MS/MS-derived protein identifications from GAPDH co-immunoprecipitation. Each protein identification listed in the table was observed in at least two of three samples; protein identifications present in negative control samples were excluded from analysis (n = 3).

### SNO-GAPDH increases mitochondrial protein S-nitrosylation

**Figure 5:**
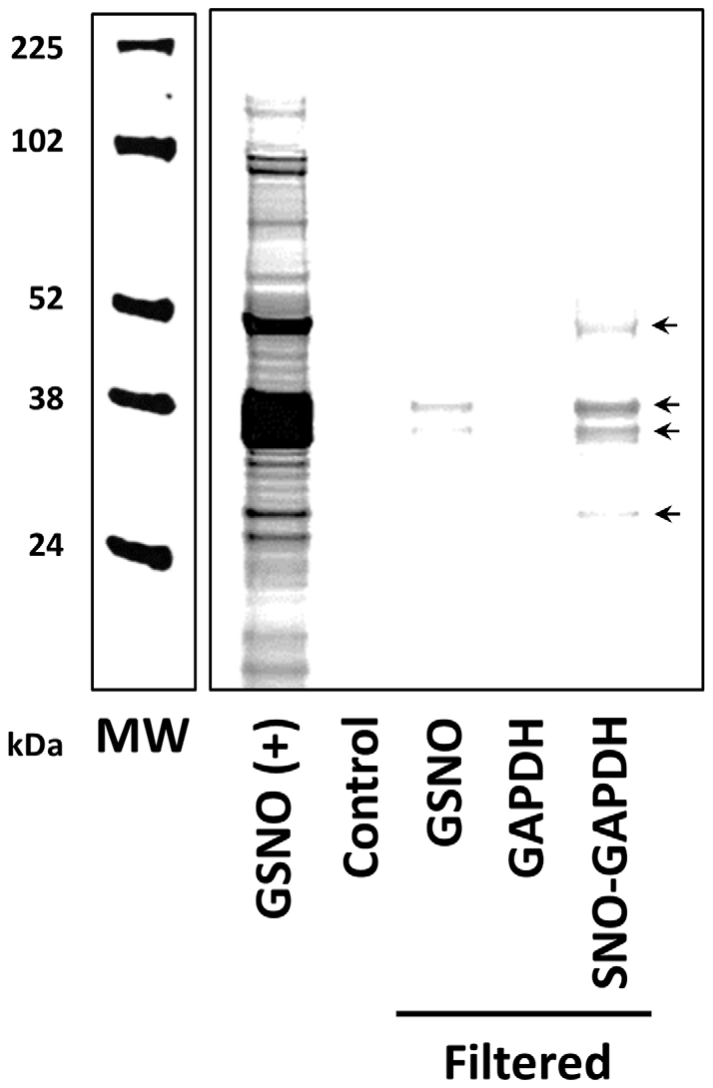
SNO-GAPDH increases mitochondrial SNO levels. A representative gel is shown for mitochondrial SNO levels measured via DyLight800 fluorescence after the addition of purified GAPDH or SNO-GAPDH. MW: molecular weight marker; GSNO (+): GSNO treatment was used as a positive control for mitochondrial SNO; Control: non-treated mitochondrial control; GSNO: filtered GSNO treated mitochondria; GAPDH: purified GAPDH treated mitochondria; SNO-GAPDH: SNO-GAPDH treated mitochondria. *Please note*: the filtration procedure serves to remove excess GSNO following the incubation of purified GAPDH with GSNO (n = 3).

GAPDH has been shown to act as a trans-*S*-nitrosylase in the nucleus [17]. Therefore, we examined the ability of GAPDH to act as a mitochondrial trans-*S*-nitrosylase. Following incubation of isolated heart mitochondria with purified SNO-GAPDH, mitochondrial SNO levels were examined using the DyLight maleimide switch method [4], and one-dimensional gel electrophoresis. Compared to GSNO alone, which elicited a large increase in mitochondrial protein SNO, the addition of SNO-GAPDH to isolated heart mitochondria elicited a modest increase in SNO for many different mitochondrial protein targets after a 30 minute incubation at room temperature, as shown in Figure 5. This was not the case when non-modified GAPDH was incubated with isolated heart mitochondria. In addition, to account for possible contaminant GSNO that was not filtered out during the process of generating SNO-GAPDH, we treated mitochondria with GSNO that had been subjected to the filtration procedure. However, this treatment induced very little change in the SNO signal. These results demonstrate the ability of SNO-GAPDH to rapidly increase mitochondrial protein SNO levels.

### GAPDH overexpression leads to an increase in mitochondrial SNO proteins

**Figure 6:**
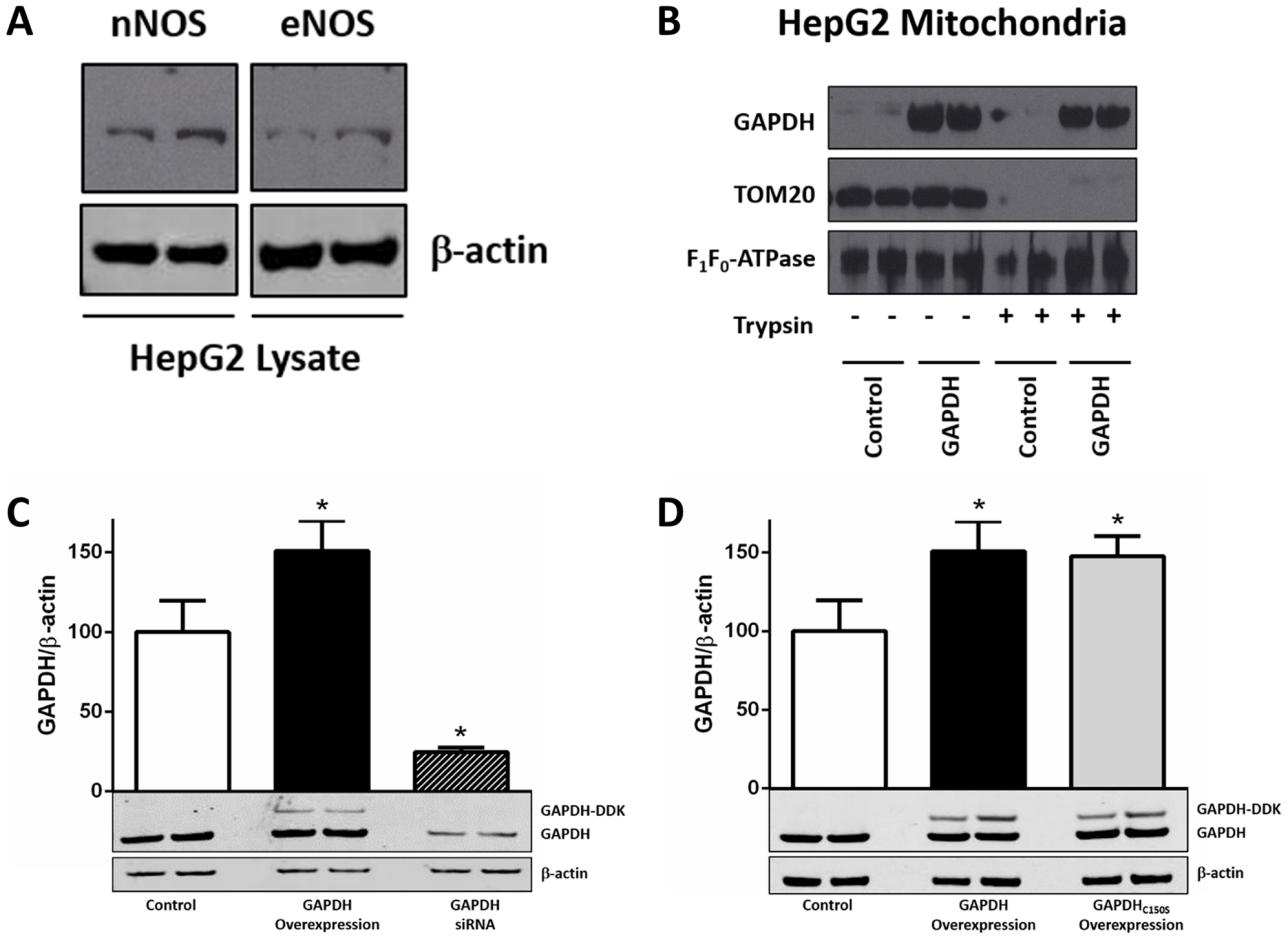
HepG2 cell line as a model system for examining GAPDH as a trans-S-nitrosylase. (**A**) Expression of neuronal and endothelial isoforms of NO synthase in HepG2 cells. Representative western blots are shown for neuronal (top right) and endothelial (top left) NO synthase and β-actin (lower). (**B**) Mitochondrial GAPDH protein levels were assessed after the addition of purified GAPDH to isolated HepG2 mitochondria. Representative western blots for GAPDH (upper), TOM20 (center), and the α subunit of F_1_F_0_-ATPase (lower) in HepG2 mitochondria. Control: non-treated mitochondrial control; GAPDH: purified GAPDH treated mitochondria; (n = 3). (**C**) and (**D**) Hep2G cells were transfected with either a control GFP plasmid or siRNA scramble, a plasmid encoding DDK-tagged GAPDH for overexpression, a GAPDH siRNA for knock-down, or a plasmid encoding DDK-tagged GAPDH_C150S_ for overexpression. Representative western blots are shown together with the densitometry of GAPDH normalized to β-actin for total GAPDH (upper; GAPDH, GAPDH-DDK, GAPDH_C150S_-DDK) and β-actin (lower; *p<0.05 vs. control; n = 6).

To further determine a role for GAPDH as a potential mitochondrial trans-*S*-nitrosylase, we utilized the SNO-RAC methodology to examine mitochondrial SNO proteins following the overexpression or knock-down of GAPDH in HepG2 cells. We chose to use the HepG2 cell line as a model system because of the high proportion of mitochondria compared to other cell lines, and the endogenous expression of both neuronal and endothelial NO synthase (Figure 6a). We first confirmed that GAPDH can be imported into HepG2 mitochondria using purified GAPDH and isolated HepG2 mitochondria (Figure 6b). A DDK-tagged variant of GAPDH was overexpressed and this increased GAPDH expression by ∼50% (Figure 6c). Conversely, siRNA-mediated knocked-down of GAPDH decreased expression to ∼25% of control levels. In addition, we utilized label-free peptide quantification to determine that SNO-GAPDH levels were increased by 62% with GAPDH overexpression compared to baseline levels, while SNO-GAPDH was below the limit of detection in siRNA-treated HepG2 cells.

**Figure 7:**
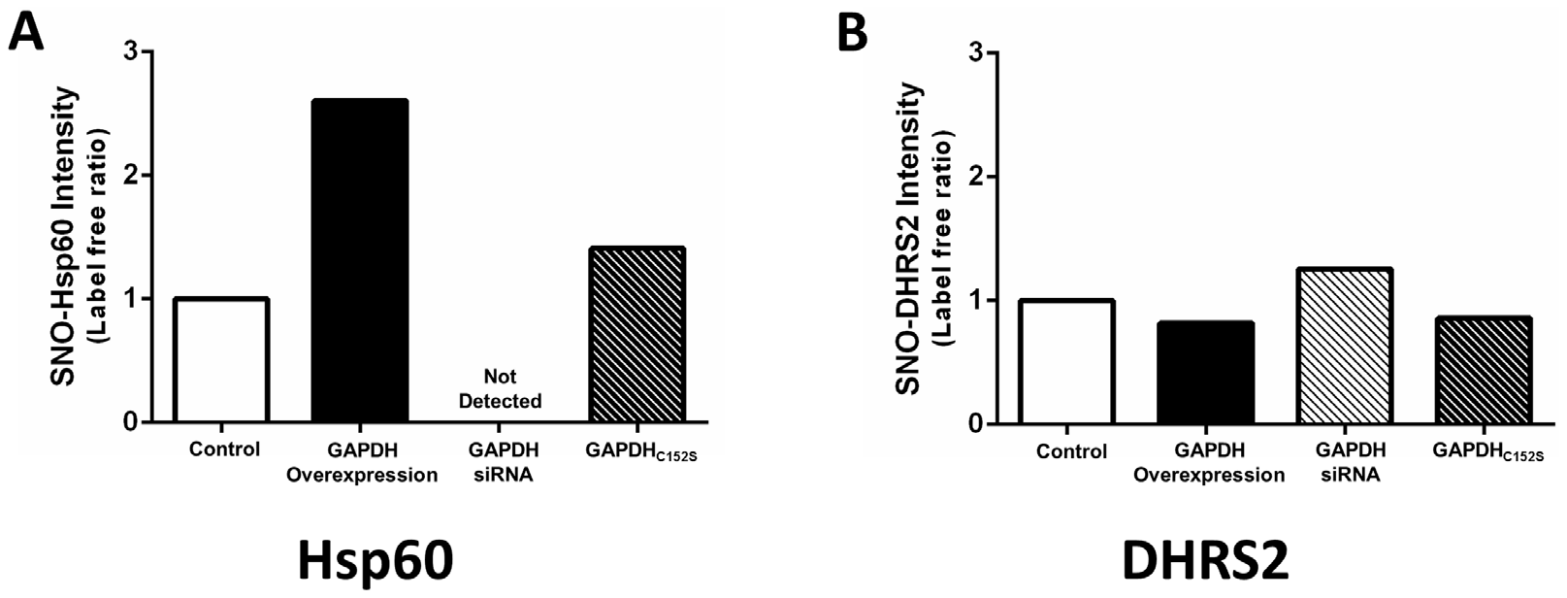
GAPDH overexpression increases in SNO-Hsp60, but not SNO-DHRS2 levels. SNO-Hsp60 (**A**) and SNO-DHRS2 (**B**) levels from Hep2G cells transfected with either a control GFP plasmid or siRNA scramble, a plasmid encoding DDK-tagged GAPDH for overexpression, a GAPDH siRNA for knock-down, or a plasmid encoding DDK-tagged GAPDH_C150S_ were assessed via SNO-RAC proteomic analysis, followed by label-free peptide quantification (n = 6).

An examination of HepG2 cell lysate with SNO-RAC revealed a number of different mitochondrial SNO proteins at baseline (Table 2), including Hsp60 (Cys442) and voltage-dependent anion channel 1 (VDAC1; Cys232). However, GAPDH knock-down induced a drastic reduction in the number of mitochondrial SNO protein identifications. The correlation between the loss of mitochondrial SNO levels and the knock-down of GAPDH suggests that these proteins may be targets for GAPDH as a trans-*S*-nitrosylase. With GAPDH overexpression, we again observed a number of different mitochondrial SNO proteins, including a new set of mitochondrial SNO proteins that were only detected with this experimental group. These proteins included acetyl-CoA acetyltransferase (ACAT1; C119) and long-chain fatty acid CoA ligase (C503). Hsp60 is another protein that is of particular interest because we have shown that Hsp60 is a common target of SNO with cardioprotection [4], and the overexpression of GAPDH led to a 2.5-fold increase in SNO-Hsp60 levels (Figure 7a). Conversely, dicarbonyl reductase (DHRS2; Cys212) is nitrosylated to the same extent in the presence of GAPDH overexpression or knock-down (Figure 7b), and is not likely to be a target of GAPDH.

**Table 2:** SNO proteins from HepG2 cells as identified via SNO-RAC proteomic analysis.

*Control*	*GADPH Knock-down*	*GAPDH_WT_ Overexpression*	*GAPDH_C150S_ Overexpression*
Dicarbonyl reductase (C212)	Dicarbonyl reductase (C212)	Dicarbonyl reductase (C212)	Dicarbonyl reductase (C212)
60 kDa heat shock protein (C442)	–	60 kDa heat shock protein (C442)	60 kDa heat shock protein (C442)
Dehydrogenase/reductase SDR family member 1 (C10)	–	Dehydrogenase/reductase SDR family member 1 (C10)	–
Voltage-dependent anion selective channel protein 1 (C232)	–	Voltage-dependent anion-selective channel protein 1 (C232)	–
Dynein light chain 1 (C24)	Dynein light chain 1 (C24)	Dynein light chain 1 (C24)	–
Voltage-dependent anion selective channel protein 2 (C47)	Voltage-dependent anion-selective channel protein 2 (C47)	–	–
–	–	Long-chain-fatty-acid – CoA ligase 3 (C503)	Long-chain-fatty-acid – CoA ligase 3 (C503)
–	–	Long-chain-fatty-acid – CoA ligase 4 (C503)	Long-chain-fatty-acid – CoA ligase 4 (C503)
–	–	Acetyl-CoA acetyltransferase (C119)	–
Leucine-rich PPR motif-containing protein (C208)	–	–	–
Pyrroline-5-carboxylate reductase 1 (C120)	–	–	–

LC-MS/MS-derived protein identifications from SNO-RAC analysis of HepG2 cell lysate. Each protein identification listed in the table was observed in at least one of six samples with at least two peptide identifications; the cysteine contained in parentheses represents the SNO modified residue; (−) indicates that SNO was not detected for a given protein identification under the specified condition (n = 6).

We also developed a GAPDH mutant that could no longer be *S*-nitrosylated at cysteine 150 (GAPDH_C150S_). The cysteine 150 residue of GAPDH has been shown to play an important role in the ability of GAPDH to act as a nuclear trans-*S*-nitrosylase [17]. With the overexpression of GAPDH_C150S_ (Figure 6d), we observed a dominant-negative effect, such that the number of mitochondrial SNO protein identifications was reduced, similar to that observed with GAPDH knock-down (Table 2). In addition, SNO-Hsp60 levels were normalized to control levels with GAPDH_C150S_ overexpression, compared to overexpression of wild-type GAPDH (Figure 6a). We also no longer observed SNO of ACAT1 as we did with the overexpression of wild-type GAPDH (Table 2). These results indicate that Hsp60 and ACAT1 have the potential to be major targets of GAPDH as a trans-*S*-nitrosylase in the mitochondria. However, both isoforms of long-chain fatty acid CoA ligase remained modified with the overexpression of GAPDH_C150S_, and this may indicate that GAPDH has additional cysteine residues that are capable of inducing trans-*S*-nitrosylation. Although GAPDH does not appear to be responsible for the trans-*S*-nitrosylation of all mitochondrial proteins, these results support the role of GAPDH as a trans-*S*-nitrosylase for specific mitochondrial protein targets.

### Consensus motif for GAPDH as a trans-S-nitrosylase

A recent study from Dr. Solomon Snyder's group suggests that a specific consensus motif may dictate the interaction of GAPDH with various target proteins, as the nuclear trans-*S*-nitrosylase activity of GAPDH was abrogated following mutation of cysteine 150 or threonine 152 [17]. These findings, in tandem with our results demonstrating a potential change in GAPDH binding with SNO (Figure 4a), suggest that a binding interaction may be necessary for GAPDH to function as a trans-*S*-nitrosylase. Therefore, we examined our SNO-RAC peptide identifications from HepG2 lysates in an attempt to identify a potential consensus trans-*S*-nitrosylation motif for GAPDH. Motif-X (http://motif-x.med.harvard.edu) was used for consensus motif analysis [25]. However, the analysis did not yield a significant consensus motif for GAPDH trans-*S*-nitrosylation. This is likely due to the small number of peptide identifications, and the possibility that some of the targets may be indirectly modified.

## Discussion

**Figure 8:**
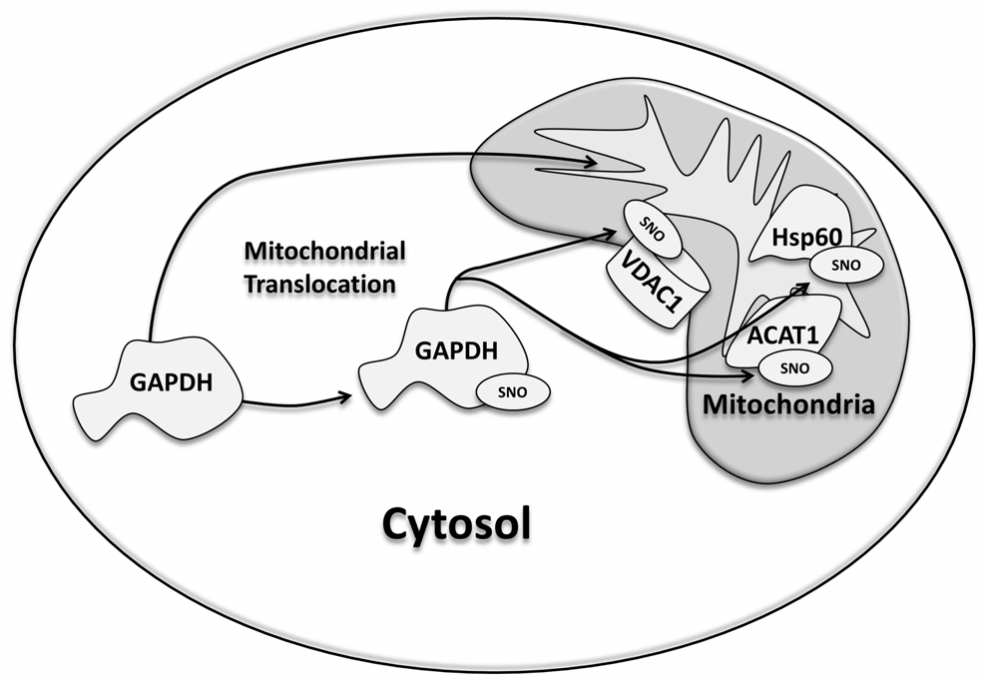
GAPDH acts as a mitochondrial trans-S-nitrosylase. GAPDH and SNO-GAPDH can be imported into the mitochondria, where SNO-GAPDH targets Hsp60, ACAT1, and VDAC1 as a mitochondrial trans-*S*-nitrosylase.

Mitochondrial proteins are major targets of SNO signaling, as our previous studies have demonstrated [3], [4], [13], [20], [21], and although the source for NO within the mitochondria remains controversial [15], trans-*S*-nitrosylases represent a potential source for the import of SNO into the mitochondria. In the current study, we are the first to demonstrate that GAPDH can act as a mitochondrial trans-*S*-nitrosylase, thus providing a foundation for the mitochondrial transmission of the SNO signal and yielding additional insight into the compartmentalization of SNO signaling. Utilizing a mass spectrometry-based approach and label-free peptide quantification, we demonstrated that GAPDH levels were increased in the mitochondrial fraction of perfused hearts subjected to IPC-IR, which is known to increase SNO-GAPDH levels [3], without a concomitant change in total GAPDH levels (Figure 2). These results suggest that the increase in mitochondrial GAPDH levels with IPC-IR is not due to changes in protein expression, but instead due to the mitochondrial translocation of GAPDH. These findings support the role of GAPDH as a mitochondrial trans-*S*-nitrosylase, and are consistent with the IPC-induced increase in mitochondrial SNO levels observed in previous studies. We further demonstrated that SNO-GAPDH can enter the matrix of control or IPC heart mitochondria (Figure 3), and our co-immunoprecipitation experiments revealed an increased interaction between SNO-GAPDH and new mitochondrial protein targets (Table 1), including aconitate hydratase, OPA1, and the mitochondrial phosphate carrier. In addition, SNO may also change the binding of GAPDH to some mitochondrial protein targets, but not all (Figure 4). Taken together, these results suggest that GAPDH remains tethered to cytoskeletal components under control conditions, which is consistent with a role for GAPDH in the regulation of cytoskeletal dynamics [26], [27], while SNO-GAPDH interacts with new protein targets, many of which are located in the mitochondria, and this leads to trans-*S*-nitrosylation. Indeed this appears to be the case, as the addition of SNO-GAPDH to isolated mitochondria led to an increase in mitochondrial SNO-proteins (Figure 5). Additionally, our use of SNO-RAC in a cell-based model (Figure 6) revealed a number of different mitochondrial SNO proteins that are targets of GAPDH as a trans-*S*-nitrosylase (Table 2), including VDAC1, ACAT1, and Hsp60, which have all been shown to be modified in the setting of cardioprotection [3], [4]. The effect of GAPDH on mitochondrial SNO protein levels could be blocked with GAPDH knock-down, thus pointing to a critical role for GAPDH in the maintenance of mitochondrial SNO levels. Overexpression of a GAPDH mutant that could no longer be modified at C150 also abrogated the effect of GAPDH on mitochondrial SNO protein levels. Interestingly, we detected an interaction between GAPDH and Hsp60 in our co-immunoprecipitation experiments (Table 1), and this interaction led to a robust increase in SNO-Hsp60 levels (Figure 7), suggesting that GAPDH may be an Hsp60 trans-*S*-nitrosylase. This interaction between GAPDH and Hsp60 does not appear to be affected by SNO (Figure 4), however, and therefore it is interesting to speculate that this interaction may play a role in the mitochondrial import of GAPDH and SNO-GAPDH. Additional experiments are required to determine the significance of this interaction. Although GAPDH does not appear to be responsible for the trans-*S*-nitrosylation of all mitochondrial proteins, as evidenced by the lack of change in SNO-DHRS2 levels (Figure 7), GAPDH appears capable of targeting specific proteins in the mitochondria (Figure 8). This is consistent with the role of GAPDH as a nuclear trans-*S*-nitrosylase, where only three nuclear protein targets were identified [17]. In addition, it is also important to consider that some of the *S*-nitrosylated mitochondrial proteins identified in this study may not be direct targets of SNO-GAPDH, but rather indirect targets, therefore leaving the potential for additional trans-*S*-nitrosylases in the mitochondria.

### Physiological ramifications of mitochondrial SNO-GAPDH

In addition to playing a role in glycolysis, GAPDH has been shown to be involved in many different cellular processes, including DNA repair [28] and cytoskeletal dynamics [26], [27]. In the current study, we describe another role for GAPDH as a mitochondrial trans-*S*-nitrosylase. Since SNO has been shown to modulate the activity of specific mitochondrial protein targets, GAPDH has the potential to play a role in the regulation of mitochondrial function during normal physiology and with cardioprotection. However, the specific role that GAPDH plays in the mitochondria remains to be determined. There are reports which suggest that the association of GAPDH with the mitochondria promotes cell death [19], [29], while other studies support the role of mitochondrial GAPDH as a pro-survival factor [18], [30]. However, it is important to note that many of the studies showing a connection between mitochondrial GAPDH and cell death were conducted using cultured cells with altered GAPDH expression. In any case, the mitochondrial targets of GAPDH may ultimately determine the functional outcome. The interaction of GAPDH with VDAC1 is consistent with a previous study [19], but the effect of SNO on the function of VDAC1 has not been examined. SNO has been shown to inhibit the ATPase activity of heat shock protein 90, and this inhibition serves as a feedback mechanism for restricting the activity of endothelial NO synthase [31]. However, the effect of SNO on Hsp60 has not been investigated. The effect of SNO on the function of ACAT1 is also unknown, but the interaction between GAPDH and ACAT1 represents a potential avenue for crosstalk between SNO and metabolism in the mitochondria.

### Conclusions

In summary, this study provides insight into a potential role for GAPDH as mediator of SNO transport into the mitochondria during normal physiology and with cardioprotection, thus offering a better understanding of the selective targeting and compartmentalization of SNO by trans-*S*-nitrosylases. The mitochondrial import of GAPDH allows this glycolytic protein to interact with mitochondrial proteins and act as a trans-*S*-nitrosylase for specific mitochondrial protein targets. These protein targets include VDAC1, ACAT1, and Hsp60. Thus, GAPDH may play a critical role in the regulation of mitochondrial function and cardioprotection by acting as a mitochondrial trans-*S*-nitrosylase.
